# Salinomycin inhibits hepatocellular carcinoma cell invasion and migration through JNK/JunD pathway-mediated MMP9 expression

**DOI:** 10.3892/or.2014.3680

**Published:** 2014-12-18

**Authors:** LING XU, TING WANG, WEN-YING MENG, JUE WEI, JIA-LI MA, MIN SHI, YU-GANG WANG

**Affiliations:** Department of Gastroenterology, Shanghai Tongren Hospital, Affiliated to Shanghai Jiao Tong University School of Medicine, Shanghai 200336, P.R. China

**Keywords:** salinomycin, hepatocellular carcinoma, invasion, migration

## Abstract

The antibiotic salinomycin (Salin) was recently identified as an antitumor drug for the treatment of several types of solid tumors. However, the effects of Salin on the migratory and invasive properties of hepatocellular carcinoma (HCC) cells are unclear. The present study aimed to determine the antitumor efficacy and mechanism of Salin in HCC cells. Human HCC cells (HCCLM3) treated with Salin showed a concentration-dependent reduction in cell migration and invasion, and this was associated with reduced MMP9 expression. The MMP9 promoter and enhancer in a luciferase reporter assay revealed that Salin can regulate MMP9 expression through an activator protein (AP-1) site within the MMP9 enhancer. JunD, one of the AP-1 components, was significantly decreased by Salin in a concentration- and time-dependent manner. Salin was able to induce c-Jun NH2-kinase (JNK) phosphorylation and to block both JunD and MMP9 expression. Our results showed that JNK phosphorylation and JunD may be involved in the Salin-regulated MMP9 signaling pathway in HCCLM3 cells and may mediate HCC cell biological characteristics. Our studies provide new insight into the antitumor effects of Salin.

## Introduction

Hepatocellular carcinoma (HCC) is a major health issue and has one of the highest mortality rates in the world (1). Unfortunately, most cases of HCC are often diagnosed at an advanced stage and are not suitable for curative treatments such as resection, transplantation (2,3), radiofrequency ablation, transarterial chemoembolization (TACE), or targeting drugs such as sorafenib (4,5). It is known that tumorigenesis is a multi-stage complex process involving multiple genes. Activation of resistant genes or the mutation of sensitive genes in the development of cancer may lead to the failure of chemotherapeutic agents. Therefore, the study of anticancer drugs is currently a hot research topic.

Salinomycin (Salin) has been widely used in animal husbandry for many years worldwide. Salin is a polyether antibiotic used to kill gram-positive bacteria including mycobacteria and parasites such as *Plasmodium falciparum*. In addition, Salin, as an ionophore with strict selectivity for alkali ions, exhibits a wide range of biological activities, including inhibition of adipogenesis and anti-allergic activity (6,7). Recent studies indicate that Salin has antitumor effects, with attenuation of proliferation, autophagy and cell death/apoptosis in human cancer cells or cancer stem cells (8–11). However, the effects of Salin on the migratory and invasive properties of HCC cells, and the underlying molecular mechanisms remain obscure.

In the present study, we demonstrated that the anti-invasive and anti-migratory effects of Salin are mediated by downregulation of MMP9 through the JNK/JunD pathway leading to inhibition of HCC cell invasion and metastasis.

## Materials and methods

### Cell lines and culture conditions

The human HCC cell lines with highly invasive capacities (HCCLM3 and MHCC-97H) were obtained from the Chinese Academy of Sciences Committee Type Culture Collection cell bank. The cells were grown in Dulbecco’s modified Eagle’s medium (DMEM; Life Technologies, Ann Arbor, MI, USA) supplemented with 10% fetal bovine serum (FBS; Life Technologies) and both penicillin and streptomycin (100 mg/ml each) at 37°C in a humidified atmosphere of 5% CO_2_.

### Cell migration and invasion assays

Cells (5×10^4^) were seeded in the upper chamber of Transwell plates with 8-μm pores (Costar, Cambridge, MA, USA). The lower chambers of the Transwell plates were filled with 500 μl medium containing 10% FBS as a chemoattractant. The plates were incubated at 37°C for 12 h. Cell invasion assays were performed using the same method. The Transwell chambers were covered with 50 μl 1:2 Matrigel and phosphate-buffered Saline mixture, and the cells were cultivated for 24 h. Cells that migrated or invaded to the lower surface were stained with Giemsa solution and quantified by counting five randomly selected microscopic fields at ×200 magnification.

### Angiogenesis assay

The supernatant was collected from the HCCLM3 cells cultured in a serum-free medium with or without Salin treatment. The Matrigel angiogenesis assay was performed as previously described (12,13). In brief, BD Matrigel (BD Biosciences San Jose, CA, USA) matrix was plated in 96-well flat-bottom cell culture cluster plates. After incubation for 30 min, 10,000 HUVEC cells/well and 50 μl of the HCCLM3 supernatant with or without Salin treatment were placed on the Matrigel. The plate was incubated at 37°C for 16–18 h. Following incubation, the wells were photographed, and the results were quantified by measuring the length of the tube-like structures using Nikon NIS-Elements computer software.

### Enzyme-linked immunosorbent assays (ELISA)

To measure the concentrations of MMP2 and MMP9 secreted from the cultured tumor cell lines, the supernatants were assessed using ELISA. Cells (5×10^4^/well) in 24-well plates were incubated at 37°C in a 5% CO_2_ atmosphere in DMEM containing 10% FBS. After 24 h, the cells were washed and incubated for 24 h in serum-free medium without/with Salin (5–20 μmol/l). The culture-conditioned medium was collected, centrifuged, and the concentrations of MMP2 and MMP9 were determined by quantitative ELISA (R&D Systems, Minneapolis, MN, USA), according to the manufacturer’s instructions.

### MMP9 promoter and enhancer and luciferase reporter gene constructs

To clone the putative promoter and/or enhancer region of the MMP9 gene, a PCR-based method was used, and specific primers were designed from the 5′-end of the known MMP9 promoter sequence from a previous study (14). The amplified DNA fragment of 2302 bp was cloned into the pGL3-Basic vector (Promega, Madison, WI, USA) to construct pGL3-MMP9-WT containing the potential enhancer element. AP-1 site-mutated MMP9 (pGL3-MMP9-mAP1-1, pGL3-MMP9-mAP1-2 and pGL3-MMP9-mAP1-1+2), NF-κB site-mutated MMP9 and Sp-1 site-mutated MMP9 (pGL3-MMP9-mSp1) luciferase promoters were used in the transient transfection assays as previously described (15,16). These vectors contain the luciferase gene driven by the SV40 promoter. The composition of the constructs was confirmed by restriction endonuclease digestion and DNA sequencing.

### Transfection and luciferase reporter assays

Cells (5×10^4^/well) in 24-well plates were incubated at 37°C in a 5% CO_2_ atmosphere in serum-free DMEM. After 24 h, the plasmids were transfected into cells using Lipofectamine-2000 reagent according to the manufacturer’s instructions (Invitrogen, Carlsbad, CA, USA). After transfection, the cells were cultured in FBS without or with Salin (5–20 μmol/l) for 24 h. The cell lysate was used to detect luciferase activity according to the manufacturer’s protocol (Promega). Luciferase activity was normalized to the β-galactosidase activity in the cell lysates and expressed as the average of three independent experiments.

### Western blotting

Total cellular proteins were extracted using RIPA buffer and quantified by the BCA method. The sample proteins were separated by electrophoresis on SDS-PAGE and transferred onto a PVDF membrane. After blocking, the membranes were incubated overnight at 4°C with various primary antibodies and α-tubulin at 1:500–1:1000 dilution (Santa Cruz Biotechnology, Santa Cruz, CA, USA). The secondary HRP-conjugated antibodies were diluted 1:2000. The immunocomplexes were detected using the ECL system (Beyotime Biotechnology, China). A Li-COR Odyssey scanner (LICOR) was used to detect the antigens on the blots.

### Statistical analysis

All statistical analyses were performed using SPSS 17.0 software. Data are reported as mean ± SD, and mean values were compared using the Student’s t-test and ANOVA. Values of p<0.05 and p<0.01 were considered statistically significant.

## Results

### Salinomycin inhibits HCC cell migration and invasion

**Figure 1:**
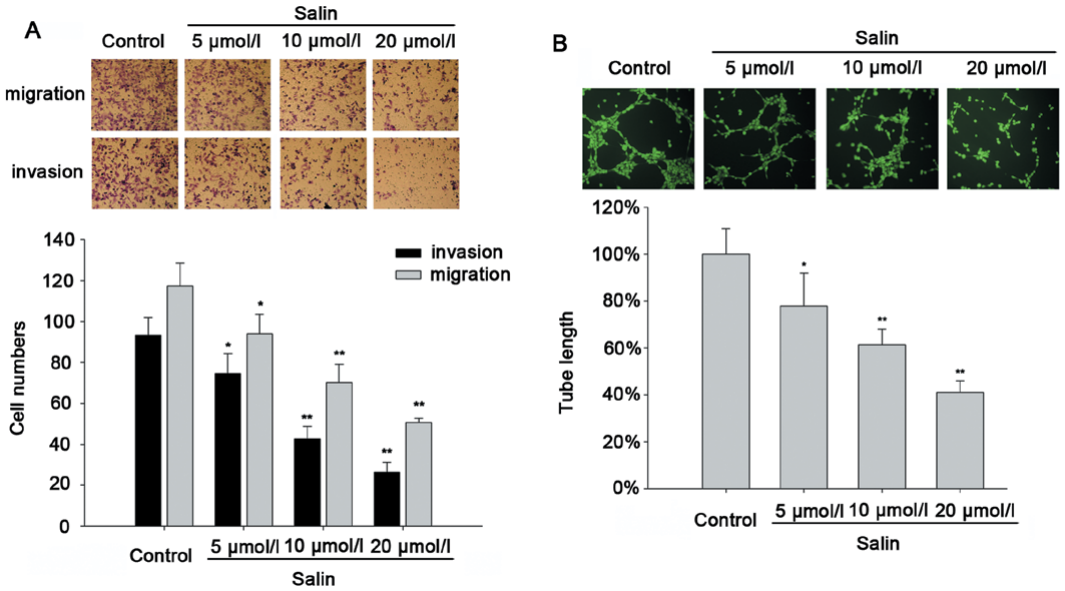
Salinomycin (Salin) inhibits cancer cell migration and invasion in HCCLM3 cells. (A) The Transwell assay indicates the inhibitory effects of Salin on the migration and invasion of the HCCLM3 cells incubated withour or with the indicated concentrations of Salin; cells were stained and counted using light microscopy (magnification, ×200). (B) The tube formation assay was used to determine the ability of HUVECs to form capillaries when cultured with the supernatant from HCCLM3 cells treated without or with the indicated concentrations of Salin. The ability of HUVECs to form capillaries was significantly impaired when they were cultured with the supernatant collected from the cells treated with different concentrations of Salin. These results are representative of three independent experiments. ^*^p<0.05 vs. the control, ^**^p<0.01 vs. the control.

It is known that Salin has anticancer activity, and previous studies have shown that it can induce cell apoptosis and suppress cancer cell proliferation. We determined the effects of Salin on the migration and invasion of HCC cells using the Transwell assay and tube formation assay. Salin (5–20 μmol/l) significantly inhibited HCCLM3 cell migration and invasion in a concentration-dependent manner (Fig. 1A). In addition, significantly fewer integrated capillary-like structures were found in the HCCLM3 cells treated with Salin (5–20 μmol/l), indicating that Salin affected the release of pro-angiogenic proteins in the HCCLM3 cells (Fig. 1B). Similar results were confirmed in the other invasive HCC cell line, MHCC-97H (data not shown), suggesting that the anti-invasive or anti-migratory effect of Salin in the HCC cell lines is universal.

### Suppression of cancer cell invasion and metastasis by Salin is associated with the downregulation of MMP9

**Figure 2:**
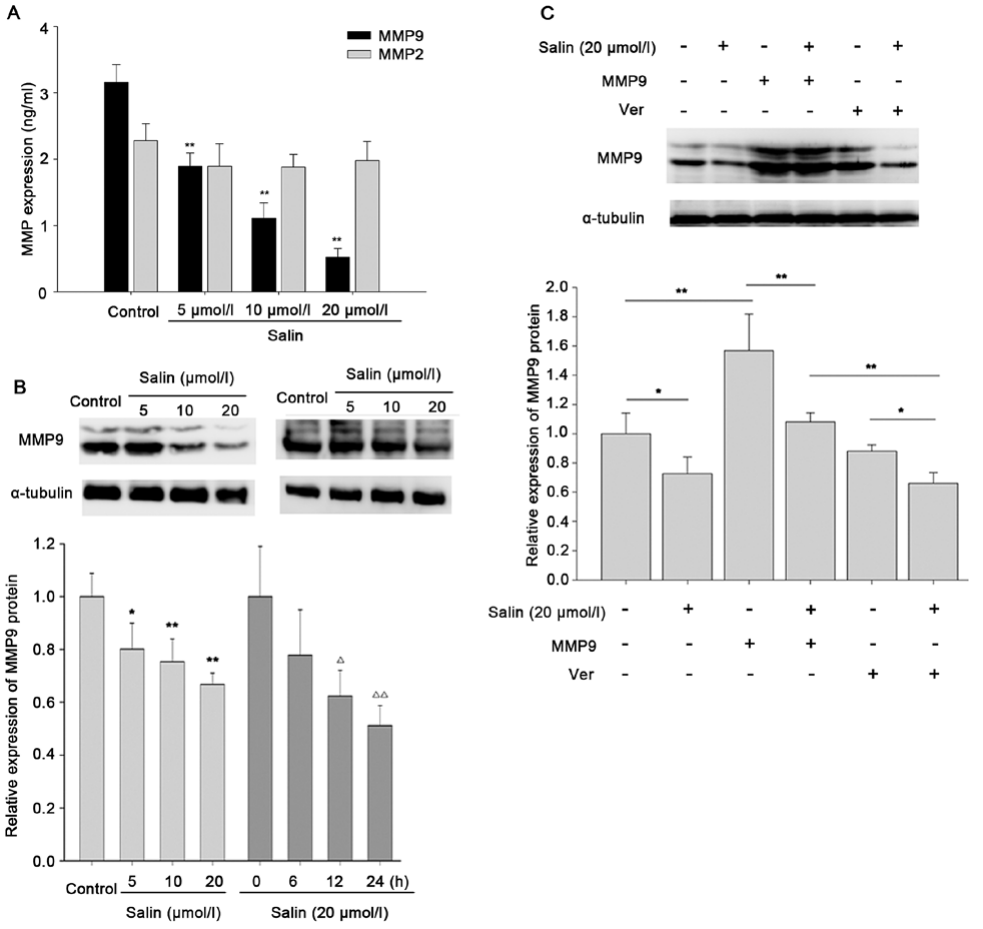
Salinomycin (Salin) inhibits MMP9 expression in the HCC cell line, HCCLM3. (A) ELISA revealed that MMP9 expression was inhibited by Salin in a concentration-dependent manner (5–20 μmol/l) after a 24-h incubation. ^*^p<0.05 vs. the control, ^**^p<0.01 vs. the control. (B) Concentration-dependent and time-dependent inhibition of MMP9 expression at the protein level by Salin were confirmed by western blot analysis. The results indicate that the protein level of MMP9 was significantly increased after Salin (20 μmol/l) treatment for 6–24 h. ^*^p<0.05 vs. the control or treated for 0 h, ^**^p<0.01 vs. the control or treated for 0 h. (C) Western blotting indicates the MMP9 expression levels in the MMP9-transfectant HCCLM3 cells with or without Salin treatment. Random group is the Ver transfectant HCCLM3 cells. ^*^p<0.05 vs. the control or random group, ^**^p<0.01 vs. the control or random group. Data are representative of at least three independent experiments.

To elucidate the anti-invasive and anti-migratory mechanisms of Salin, we examined MMP9 and MMP2 expression. HCCLM3 cells were treated with Salin at concentrations of 5–20 μmol/l. The results showed that Salin at 5 μmol/l or higher concentrations attenuated MMP9 expression in the HCCLM3 cells, but did not significantly affect the expression of MMP2 (Fig. 2A). In addition, Salin inhibited MMP9 expression at the protein level in a concentration-dependent and time-dependent manner (Fig. 2B). To determine the importance of MMP9 in the anti-invasive effect of Salin in HCCLM3 cells, we established stable overexpression of MMP9 in the HCCLM3 transfectants by infecting the cells with a lentivirus encoding MMP9. The results showed that overexpression of MMP9 was significantly blocked by 10–39% in the Salin-treated group compared with the group without Salin treatment (p<0.01; Fig. 2C). The results also showed that Salin decreased MMP9 expression by 14–36% compared with the random group (Ver transfectants) (p<0.05; Fig. 2C). These results suggest that MMP9 may play a role in the anti-invasive and anti-migratory effects of Salin.

### The AP-1 site within the MMP9 enhancer is essential for Salin-regulated MMP9 activity

**Figure 3:**
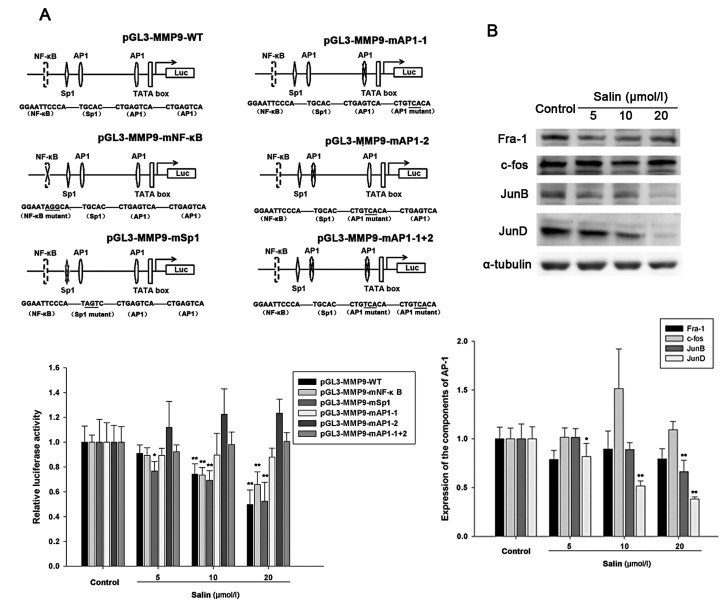
Salin inhibits MMP9 expression by decreasing JunD and AP-1 activation. (A) The regulation of Salin on the MMP9 promoter and enhancer. Relative luciferase activities of pGL3-MMP9-WT (containing the potential enhancer element) and several mutant derivatives of the MMP9 enhancer region for all motifs (pGL3-MMP9-mNF-κB, pGL3-MMP9-mSp1, pGL3-MMP9-mAP1-1, pGL3-MMP9-mAP1-2 and pGL3-MMP9-mAP1-1+2) were transfected in HCCLM3 cells treated without or with Salin (5-20 μmol/l). The results revealed that the AP-1 site is critical for Salin-regulated MMP9 enhancer activity. (B) Western blot analysis indicated that the expression of the components of AP-1 (JunB and JunD) was regulated by Salin (5–20 μmol/l). ^*^p<0.05 vs. the control, ^**^p<0.01 vs. the control. These results are representative of three independent experiments.

The MMP9 promoter contains two AP-1 sites (located at −79 bp and −533 bp), a Sp1 site (located at −560 bp) and an NF-κB site (located at −600 bp). To determine whether regulation of MMP9 is related to these *cis*-acting regulatory elements for transcription factors, several constructs with deletions or mutations were used and have been described in Materials and methods. Experimental cells were transfected with reporter vectors that included the tandem repeat of AP-1, NF-κB or Sp-1 binding sites. Noteworthy, luciferase activity in the cells with the AP-1 construct was significantly reduced by treatment with Salin at 10–20 μmol/l, whereas luciferase activity containing the NF-κB or Sp-1 binding site construct showed no statistically significant changes in the cells treated with Salin (Fig. 3A). These results showed that both AP-1 sites in the MMP9 promoter were essential for Salin-regulated MMP9 enhancer region activity.

### JNK/JunD signaling pathway is responsible for MMP9 downregulation by Salin

The AP-1 complex is composed of several protein components, including c-Fos, JunD, JunB and Fra-1. We aimed to determine which members play a major role in the downregulation of MMP9 by Salin. The results demonstrated that only JunD was significantly downregulated by Salin in the HCCLM3 cells (Fig. 3B). JunB showed a slightly increasing trend, particularly following treatment with 20 μmol/l Salin, whereas Fra-1 and c-Fos were not significantly affected by Salin at concentrations ranging from 5 to 20 μmol/l (Fig. 3B).

**Figure 4:**
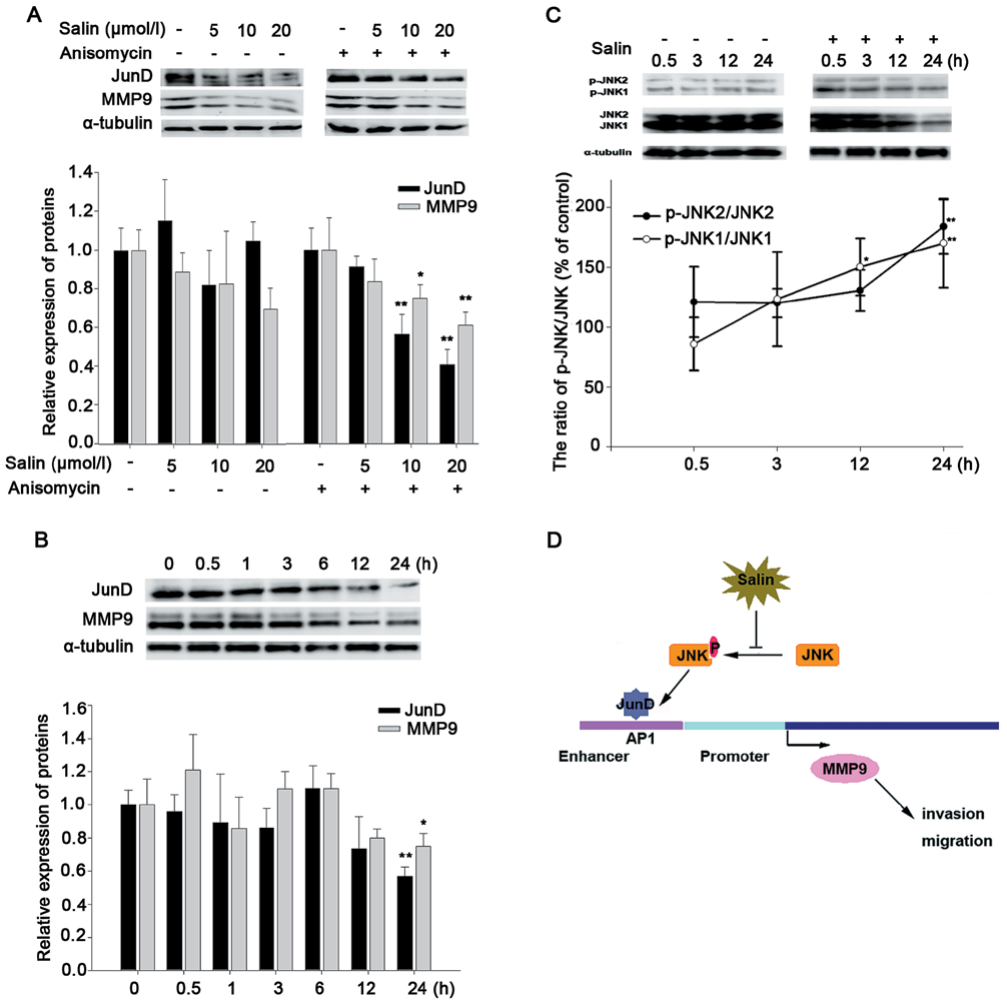
JNK/JunD signaling pathway is involved in MMP9 downregulation by Salin. (A) Salin-regulated JunD and MMP9 expression. Western blotting showed that Salin inhibited JunD and MMP9 expression induced by anisomycin, a JNK activator. (B) Western blotting revealed that JunD and MMP9 expression was inhibited by Salin at the indicated times (0, 0.5, 1, 3, 6, 12 and 24 h) during incubation. (C) The effects of Salin on JNK activation at the indicated time periods (0.5, 3, 12 and 24 h). After treatment without or with 20 μmol/l Salin, JNK1, JNK2, p-JNK1 and p-JNK2 were examined by western blotting with JNK and phosphor-JNK specific antibodies. The ratios of p-JNK1/JNK1 and p-JNK2/JNK2 showed that JNK phosphorylation was regulated by Salin. (D) The hypothetical mechanism by which Salin causes MMP9 inhibition and related signaling events in the presence of its anti-invasive and anti-migratory effects. ^*^p<0.05 vs. the control, ^**^p<0.01 vs. the control. Data are representative of at least three independent experiments.

To evaluate the role of JunD in Salin-regulated MMP9 activity, we investigated JunD and MMP9 expression at different times and at different Salin concentrations. The data showed that Salin simultaneously reduced JunD and MMP9 protein expression (Fig. 4A). It was also shown that Salin affected protein expression at 10 μmol/l or higher concentrations, and this was concentration-dependent (Fig. 4A). In addition, as shown in Fig. 4B, the target proteins were inhibited by Salin in a time-dependent manner. The dynamic effects of JunD and MMP9 expression occurred after 12 h.

To further confirm the effects of Salin on these signaling cascades, anisomycin, an activator of c-Jun N-terminal kinase (JNK), was added to the HCCLM3 cells with or without Salin. The results showed that Salin blocked anisomycin-induced JunD expression (Fig. 4A). Antibodies against the phosphorylated forms of JNK were then used to determine the changes in the phosphorylation level following Salin intervention. The data showed that the phosphorylated forms of JNK1 and JNK2, p-JNK1 and p-JNK2, were significantly impeded by Salin in a time-dependent manner (Fig. 4C). These results showed that JNK phosphorylation and JunD may be involved in the Salin-regulated MMP9 signaling pathway in HCCLM3 cells and may mediate HCC cell biological characteristics.

## Discussion

Salinomycin (Salin) is crucially involved in the regulation of cell proliferation, growth and survival, in addition to anti-tumorigenesis. However, the role of Salin in cancer invasion and metastasis is not well understood. In the prsent study, we confirmed that Salin inhibited HCC cell migration and invasion *in vitro* through the downregulation of MMP9 rather than MMP2. In addition, our results indicated that Salin regulated MMP9 at the protein level in a concentration-dependent and time-dependent manner.

It is well known that degradation of the extracellular matrix is the angiogenic switch in tumorigenesis. In particular, MMP9, its enhancer and promoter can bind to the transcription factors AP-1, Sp1 and NF-κB, and can be affected by a variety of signals (16). Several natural products or chemicals play a role in antitumor activity by interfering with MMP9 gene expression. For example, hesperidin obstructs the activity of MMP9 by inhibiting NF-κB (17), and genistein suppresses MMP9 transcription by inhibiting AP-1 and NF-κB activity (18). In the present study, we showed, for the first time, that Salin acts through AP-1 to inhibit MMP9 expression. Previous evidence indicated that the regulation of JunD expression diverges from the well-characterized growth factor-inducible pattern of the c-Jun early response genes and AP-1 autoregulation (19,20). Furthermore, we found that the onco-suppressive effects of Salin may partially mediate MMP9 expression via the JNK/JunD pathway, causing the suppression of cancer cell invasion and migration (Fig. 4D). These results suggest that Salin inhibits cancer-cell invasion by decreasing JNK/JunD signaling and AP-1 activation to prevent MMP9 expression.

In conclusion, we discovered novel pathways associated with the ability of Salin to suppress tumorigenesis. The onco-suppressive effects of Salin may be partially mediated via JNK/JunD-regulated MMP9 expression. These findings confirm the possibility that Salin may be a potential anticancer drug targeting MMP9.
